# CT radiation dose awareness among paediatricians

**DOI:** 10.1186/s13052-016-0290-3

**Published:** 2016-08-31

**Authors:** Tamader Y. AL-Rammah

**Affiliations:** Department of Radiological Sciences, College of Applied Medical Sciences, King Saud University, P.O.Box 10219, Riyadh, 11433 Kingdom of Saudi Arabia

**Keywords:** ALARA, Cancer risk, Computed tomography, Paediatrics, Radiation dose

## Abstract

**Background:**

The radiation dose delivered from computed tomography (CT) scanning and the risks associated with ionising radiation are major concerns in paediatric imaging. Compared to adults, children have increased organ sensitivity and a longer expected lifetime in which cancer may develop. Therefore, it is important to investigate the awareness of paediatricians (referring physicians) regarding radiation doses and the associated risks.

**Methods:**

A multiple-choice survey was distributed among paediatricians in 8 hospitals in Riyadh, the capital of Saudi Arabia.

**Results:**

Among the 162 respondents, only 24 (15 %) were aware of the As Low As Reasonably Achievable (ALARA) principle. Approximately half (54 %) of the respondents believed that multi-slice CT delivered a low radiation dose, and 100 (62 %) of the respondents were not aware that radiation is considered carcinogenic by the Food and Drug Administration in the United States. Among the respondents, 110 (68 %) did not have any specific education regarding radiation during their training. There was an overall underestimation (83 %) of the CT radiation dose, and 70 % thought that magnetic resonance imaging (MRI) delivered some level of ionising radiation.

**Conclusions:**

Among paediatricians in Saudi Arabian hospitals, there was a wide underestimation of the CT radiation dose and the associated risks for children. We should improve paediatricians’ knowledge about radiation doses. Radiologists, paediatricians, radiation technologists and medical physicists should work together to optimise CT guidelines and protocols to reduce the radiation risks for children.

## Background

The introduction of computed tomography (CT) in 1972 added a valuable diagnostic technique to medicine. With the rapid developments and advances in CT technology, both the number and variety of its applications have dramatically increased.

Countries with high health standards had an eight-fold increase in CT usage since the introduction of CT in the 1970s to the mid-1990s [1–3]. This fact has raised concerns about possible cancer risks, particularly after exposure during childhood. CT accounts for approximately 24 % of total radiation exposure and 49 % of the total medical imaging. Therefore, CT scans are a major source of radiation received by patients [4]. CT examinations account for only 15 % of the total number of medical diagnostic procedures but over half of the collective dose with dental scans excluded [5]. It is estimated that in the United States alone there are more than 62 million CT scans performed per year, and 4 million CT scans are performed on children [6].

These facts have raised concerns about exposing children to medical radiation, and many publications have discussed paediatric radiation issues [7–10]. Pearce et al, work showed significant association between the estimated radiation doses provided by CT scan to red bone marrow and brain, and subsequent incidence of leukaemia and brain tumours [11]. On the other hand, deep knowledge of radiation risk is mandatory for referring physicians to evaluate the risk against benefit of different CT procedures. Thus, it is important to investigate the level of radiation knowledge among paediatricians requesting CT examinations. Several studies have investigated the level of effective radiation and dose awareness among paediatricians in different countries [12–15]. To our knowledge, no studies have been conducted in Saudi Arabia.

This study investigated the level of knowledge and awareness of the radiation dose and risks associated with radiological investigations in children among a sample of paediatricians in Saudi hospitals.

## Methods

The medical research ethics committee in our institution has approved the study. In total, 180 survey sheets with 15 questions were distributed to paediatric physicians and surgeons. One hundred sixty-two surveys were completed and returned from 8 hospitals (two university hospitals, two private hospitals and four government hospitals). The respondents were in charge of managing paediatric patients in medical or surgical specialties or in general paediatric medicine and were responsible for requesting CT examinations. For simplicity, we here refer to the entire group as “paediatricians”.

The questionnaire [16] was multiple choice and was divided into three sections. The first section obtained demographic information. The second section evaluated the participant’s basic knowledge of the fundamentals of ionising radiation. The third section assessed the participant’s ability to estimate the radiation dose delivered during common radiation procedures. This ability was assessed by asking the participant to consider a posterior-anterior chest x-ray of a 5-year-old child (0.006 mSv) as 1 unit. Paediatricians participating in this study were asked to estimate the effective dose of some common radiological procedures compared with the number of chest x-ray equivalents. This method is considered user friendly and was previously reported [16].

The effective dose was defined as the sum of the weighted equivalent of radiation doses in all organs and tissues of the body (each weighted according to their radiation sensitivity).

### Statistical analyses

The data were analysed using commercially available software Statistical Package for Social Sciences, version 23 SPSS Inc., Chicago, IL, USA).

The statistical analyses were performed using Chi-square tests. The means of the continuous measurements were compared by independent sample t-tests. The normality of the continuous variables was assessed prior to the application of parametric methods. A *p*-value >0.05 was considered significant. All descriptive data were reported as percentages.

## Results

Of the 180 surveys, 162 were completed and returned (90 % response rate). In total, 63 (39 %), 80 (50 %), and 19 (11 %) responses were from university, government, and private hospitals, respectively. Ninety-four (58 %) of the respondents were specialised paediatricians (consultants), and 68 (42 %) were paediatric residents. Regarding the respondents’ experience, 41 (25 %) had <5 years of experience, 23 (14 %) had 5-10 years of experience, 65 (40-1 %) had 11-20 years of experience and 33 (21 %) had more than 30 years of experience (Table 1).

**Table 1:** Respondents’ clinical experience based on hospital distribution

	University Hospital	Government Hospital	Private Hospital	Total
Less than 5 years	25	13	3	41
5-10 years	6	16	1	23
11-20 years	18	37	10	65
More than 20 years	14	14	5	33
Total	63	80	19	162

### Knowledge of radiation protection

Among the 162 respondents, regarding the question inquiring about the percentage of background radiation caused by medical imaging, 33 (20 %) answered correctly, 16 (10 %) underestimated the value, 44 (27 %) overestimated the value, and 69 (43 %) responded, “I don’t know”. In addition, we found that only 24 (15 %) of our respondents were familiar with the As Low As Reasonably Achievable (ALARA) principle, and 138 (85 %) were not. In response to the question that estimated the excess life-time cancer risk of a 1-year-old child undergoing a CT scan, 35 (22 %) respondents gave the correct estimate, 15 (9 %) overestimated the risk, 49 (30 %) underestimated the risk, 6 (8 %) thought that there was no excess risk, and 57 (35 %) did not know.

Regarding multi-slice CT scans, 88 (54 %) respondents believed that multi-slice scanners delivered less radiation than single-slice helical scanners. Among the remaining respondents, 56 (35 %) thought multi-slice scanners delivered more radiation than single-slice scanners, and 18 (11 %) thought that the methods delivered similar radiation doses.

When the respondents were asked if they were aware that the Food and Drug Administration in the United States lists radiation as a carcinogen, 100 (62 %) participants responded that they were not aware of this fact.

### Informed consent

When asked about the frequency of discussing radiation dose with the patients’ families, 58 (36 %) never had the discussion, 59 (36 %) rarely had the discussion, 61 (38 %) could not remember the last time they were asked about radiation dose, and 44 (27 %) were rarely asked about the radiation dose (1 in every 100 patients). Surprisingly, 104 (64 %) respondents thought that radiation risk should not be discussed routinely with the patients’ families prior to a CT request. Of these 104 respondents, 49 (47 %), 42 (40 %) and 13 (13 %) were from university, government and private hospitals, respectively (significant correlation, *p* < 0.05).

### Level of education

Among our respondents, 110 (68 %) did not have any specific education regarding radiation during their training. Only 12 (23 %) of the respondents had received specific education regarding radiation, and these respondents had formal education (lectures, workshops, courses, or radiology rotations). Only 11 (21 %) of the 52 who had received any specific education regarding radiation doses from medical imaging were familiar with the ALARA principle, and 97 (88 %) of the 110 respondents who did not receive any type of training were also not familiar of the ALARA principle.

### Estimation of effective dose

**Fig. 1:**
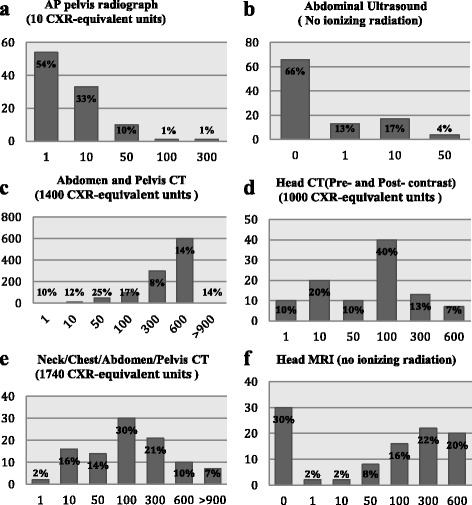
Estimation of the effective dose of a 5-year-old child during a PA chest x-ray (equivalent units). **a**) AP pelvic radiograph (10 CXR-equivalent units); **b**) abdominal ultrasound (No ionizing radiation); **c**) abdomen and pelvic CT (1300 CXR-equivalent units); **d**) head CT (Pre- and Post- contrast) (1100 CXR-equivalent units); **e**) neck/chest/abdomen/pelvic CT (1700 CXR-equivalent units); **f**) head MRI (no ionizing radiation)

In this study, a frontal (PA) chest x-ray (CXR) of a 5-year-old child was used as a standard unit, and the respondents were asked to estimate the effective dose of different imaging examinations (Table 2) In general, there was an overall underestimation of the effective dose (83 %) (Fig. 1). Only 66 % of our respondents were aware that there was no ionising radiation during abdominal ultrasounds, and 70 % of our respondents thought that a head MRI delivered some level of ionising radiation. The highest estimate was 300 times higher than a PA CXR (22 %).

**Table 2:** Reference CT effective doses and the number of CXR equivalents for a 5-year-old child (derived from our department)

Exam type	Effective dose	CXR equivalent
PA CXR	0.006	1
AP Pelvis	0.06	10
Abdomen and pelvic CT	8.4	1400
Head CT	4.12	1000
Neck/chest/abdomen/pelvic CT	10.44	1740

Regarding CT effective dose estimation, there was an overall underestimation (93 %). Only 14 % of our respondents believed that the effective dose of an abdominal and pelvic CT scan was >900 PA CXR, and 100 % of the respondents underestimated the effective dose of a head CT (pre- and post- contrast) scan. Additionally, 93 % of the respondents underestimated the effective dose of a neck/chest/abdomen/pelvic CT scan.

## Discussion

With new and rapid developments in CT technology, CT scans account for 42 % of the total effective dose arising from medical diagnostic radiology [17]. Over the last decade, there has been growing concern regarding the risk of malignancy due to ionising radiation associated with CT. Due to rapid advancements in CT technology that allow for faster scanning (therefore reducing the need for sedation or anaesthesia in young patients) and the new software programs that support additional diagnostic protocols, the rate of CT paediatric imaging has been increasing dramatically [18].

Major international organisations, such as the International Commission on Radiology Protection, the International Atomic Energy Agency and the European Commission, provide radiation education. All of these agencies have recommended the implementation of CT dose guidance levels to optimise the CT dose [19].

The three known basic fundamental principles of radiation protection are: justification, optimisation and dose limitation [20, 21]. The American College of Radiology in the United States and the Royal College of Radiology in the United Kingdom have provided guidelines that cover the referral and appropriate use of medical imaging examinations for specific clinical conditions [22, 23]. Justification is the most important factor, and the referring physician and radiologist should weigh the benefits of a specific examination against the possible risks of radiation. In addition, CT examinations should always be replaced with other non-radiating imaging methods, such as ultrasound imaging (U/S) and magnetic resonance imaging (MRI), whenever possible. Follow-up imaging should also be considered. CT scans should be limited and not performed too early. In addition, repeated scanning of the area, such as multi-phase scanning, should be carefully justified for each patient. These guidelines are the best way to reduce the radiation risk associated with diagnostic imaging [24].

Children have a greater risk of malignancy resulting from radiation than adults do. The developing tissues in children are more sensitive to ionising radiation compared to mature tissue. Additionally, children have a life time ahead of them. Therefore, their bodies have more time to manifest the oncogenic effects of ionising radiation. Adults may die from other causes before showing any significant effects from radiation.

In addition, CT scan parameters are not appropriately adjusted for paediatric patients, and the smaller cross-sectional area of a child results in a dose of radiation that is concentrated in a smaller amount of tissue, which may result in a higher effective dose compared to an adult scan [25]. A recent study found that adult CT exposure parameters were used for paediatric patients in 11 CT facilities in six countries [24].

Paediatricians play an important role in the optimal and safe application of CT scans when they refer patients for imaging. Therefore, paediatricians should be aware of the radiation risks associated with CT scans and should be able to justify a referral. In addition, paediatricians are responsible for providing families with understandable and sufficient information regarding radiation and its potential risks to help families make informed treatment decisions [26–29].

This study evaluated the level of awareness among paediatricians in Saudi Arabia regarding ionising radiation risks associated with CT scans. Our response rate was 90 % and was the range expected when designing the survey.

The majority of respondents were not aware of the contribution of radiologic imaging to background radiation (80 %). It was very disappointing that only 15 % were familiar with the ALARA principle, which was similar to the results reported by other studies. Among our respondents, 35 % did not know if there was an excess lifetime cancer risk for a child undergoing a CT scan, and 30 % of respondents underestimated the risk. Sixty-two percent of respondents were not aware that the Food and Drug Administration (FDA) in the United States lists radiation as a carcinogen, whereas 54 % believed that multi-slice CT scans delivered lower radiation doses than single-slice CT scans.

This underestimation and low level of awareness regarding ionising radiation risks and hazards is similar to the results reported in the literature [16] and may easily lead to unjustified CT referrals and unnecessary scanning of children.

It was very disappointing to find that 64 % of the participating paediatricians thought that radiation risk should not be discussed routinely with the patients’ families prior to CT requests, which probably stems from the belief that the physician knows what is most beneficial to the patient. However, parents must be clearly aware of any possible risk associated with a procedure to be able to give informed consent.

More than two-thirds of the respondents did not have any specific education regarding radiation during their training. Among the one-third of respondents who did have specific radiation education, only 21 % were familiar with the ALARA principle. This finding strongly indicates that paediatricians are poorly educated about the risks associated with ionising radiation, which can greatly influence the number of unnecessary CT referrals [30]. The guidelines of the European Community in the field (EC-Medical Exposure Directive), recommended to the member states to introduce courses on radiation protection in the basic training curriculum of surgeons and dentists and recently in all physician training [15]. University hospitals in Saudi Arabia have ongoing programs, courses, seminars and conferences on radiation protection and radiation safety. It is important to insure the enrolment of residents in these continues education activities by making them an essential component of their training program. It is also important to add such training activities to all resident programs around the country by having these activities as part of The Saudi Commission for Health Specialties licencing requirements.

For simplification, the effective dose in terms of CXR was used in this study to estimate the dose of different radiologic procedures. This method has been used previously and is effective.

The effective dose is the most useful means of assessing radiation risk to a particular body area. The effective dose is expressed in mSv and is defined by the International Commission of Radiologic Protection as the single dose quantity reflecting the overall risk to a reference person from any radiation exposure. The risk is averaged over all ages and both sexes. The effective dose for a CT scan can be calculated from the CT parameters used, including scan length, pitch, tube current and tube voltage. The dose length product (DLP) can be used to calculate the effective dose through the use of conversion factors of body region and the age of the patient [25].

There was an overall underestimation of the effective dose associated with different imaging procedures (83 %). However, 70 % of our respondents thought that MRI produced ionising radiation, and 22 % thought that this radiation was 300 times higher than CXR. Salerno et al, reported in a similar study that 21 % of the participants were not aware that MRI does not use ionizing radiation [15] and 14 % of participants in a study done on paediatricians in Germany thought the MRI uses ionizing radiation [31]. This finding is another indication of the weakness in radiation risk education among paediatricians. Sixty-six percent of the respondents were aware that there is no ionising radiation from abdominal U/S (and is widely used in obstetrics imaging). When asked specifically to estimate the CT effective dose, 93 % of respondents underestimated. 100 % of respondents underestimated the effective dose for a head (pre- and post- contrast) CT scan.

Homberg et al. reported that excessive, unnecessary exposure to patients is usually the result of medical radiological procedures that are not justified for a specified objective. Unintended patient exposure can also arise from unsafe design or use of medical technology. Radiation protection for patients requires protection from both unnecessary and unintended exposure [17].

It is important to protect paediatric patients from unnecessary ionising radiation exposure. As mentioned above, the FDA has established several guidelines for this purpose. 1) Improve exposure factors to reduce unnecessary paediatric patient radiation and perform more extensive quality checks to evaluate the reported dose values. 2) Reduce the number of procedures that require multiple CT scans. 3) Utilise alternative, low-dose radiographic exams whenever possible.

Our results strongly indicate low awareness levels of radiation risk among paediatricians practicing in Saudi Arabian hospitals, but these results are not different from similar reports from other countries around the world [16]. This report should highlight the importance of reviewing paediatric training programs to ensure that they include a sufficient amount of education regarding radiation risks. Paediatric radiologists should also have a role in monitoring hospital guidelines, including justifying referral for CT studies, optimising CT parameters and imaging protocols and educating paediatricians about new technologies and the best utilisation of medical imaging.

In 2008, the American College of Radiology (ACR), the Society of Paediatric Radiology (SPR), the American Association Physicists in Medicine (AAPM) and the American Society of Radiologic Technology (ASRT) founded the ‘Alliance for Radiation Safety in Paediatric Imaging’ as part of the Image Gently Campaign. This campaign focuses on the unique needs of paediatric patients when using ionising radiation. The aim of this alliance was to reduce the amount of ionising radiation that children are exposed to during radiological investigations. There is a great need for such a movement in Saudi Arabia and around the world to improve the overall awareness of radiation risks and hazards to children [32, 33].

## Conclusion

This study suggests that there is a widespread underestimation of the risks associated with ionising radiation among paediatricians in Saudi Arabia. It is important to ensure a sufficient and continuous level of education to physicians so they are able to weigh the risks and benefits of each radiological procedure. It is also important that paediatricians, paediatric radiologists, radiologic technologists and medical physicists work together towards optimising CT guidelines and protocols to reduce radiation exposure and the risks associated with CT scanning in children.

## References

[1] Krille L (2012). Computed tomographies and cancer risk in children: a literature overview of CT practices, risk estimations and an epidemiologic cohort study proposal. Radiat Environ Biophys.

[2] Verdun FR (2008). CT radiation dose in children: a survey to establish age-based daignostic reference levels in Switzerland. Eur Radiol.

[3] UNSCEAR (2000). Report to general assembly. Annex D: medical radiation expousure.

[4] Donnelly L (2001). Minimizing radiation dose for pediatric body applications single-detector helical CT: strategies at a large children hospital. Am J Roentgenol.

[5] Measurments, N.C.o.R.P.a (2009). Report n. 160 Ionizing radiation exposure of the population of the United States.

[6] Ogbole GI (2010). Radiation dose in paediatric computed tomography: risks and beniefits. Ann Ib Postgrad Med.

[7] Mathews JD, et al. Cancer risk in 680 000 people exposed to computed tomography scans in childhood or adolescence: data linkage study of 11 million Australians. BMJ. 2013: 346-364. 10.1136/bmj.f2360 PMC3660619 23694687

[8] Hammer GP (2010). Childhood cancer risk from conventional radiographic examinations for selected referral criteria: results from a large cohort study. Am J Roentgenol.

[9] Colagrande S (2014). CT exposure in adult and paediatric patients: a review of the mechanisms of damage, relative dose and consequent possible risks. Radiol Med.

[10] Frush DP (2011). CT dose and risk estimated in children. Pediatr Radiol.

[11] Pearce M, Salloti J, Gonzalez A (2012). Radiation exposure from CT scans in childhood and subsequent risk of leukamia and brain tumours: a retrospective cohort study. Lancet.

[12] Rice H (2007). Peer assessment od pediatric surgeons for potential risks of radiation exposure from computed tomography scans. J Pediatr Surg.

[13] Heyer CM (2010). Paediatrician awareness of radiation dose and inherent risks in chest imaging studies—a questionnaire study. Eur J Radiol.

[14] Rice HE (2007). Review of radiation risks from computed tomography: essentials for the pediatric surgeon. J Pediatr Surg.

[15] Salerrno S (2015). Radiation risks knowledge in resident and fellow in paediatrics: a questionnaie survey. Ital J Pediatr.

[16] Thomas KE (2006). Assessment of radiation dose awarness among pediatricians. Pediatr Radiol.

[17] Holmberg O (2010). Current issues and actions in radiation protection of patients. Eur J Radiol.

[18] Shah NB, Platt SL (2008). ALARA: is there a cause for alarm? Reducing radiation risks from computed tomography scanning in children. Curr Opin Periatr.

[19] Al Mohiy H (2012). A dose comparison survey in CT departments of dedicated paediatric hospitals in Australia and Saudi Arabia. World J Radiol.

[20] Young C, Owens C (2013). Pediatric computed tomography imaging guideline. Acta Radiol.

[21] Frush DP (2011). Justification and optimization of CT in children: how are we performing?. Pediatr Radiol.

[22] Sodhi KS, EY L (2014). What all physicians should know about the potential radiation risk that computed tomography poses for paediatric patients. Acta Paediatr.

[23] Nader G (2006). Pediatric CT scan usage in Japan: results of a hospital survey. Radiat Med.

[24] Khong P, Frush D, Ringertz H. Radiological protection in paediatric computed tomography. Ann ICRP. 2011;41:170-178. 10.1016/j.icrp.2012.06.017 23089016

[25] Livingston MH (2014). Radiation from CT scans in paediatric trauma patients: indications, effective dose, and impact on surgical decisions. Injury.

[26] Jacob K, Vivian G, Steel J (2004). X-ray dose training: are we exposed to enough?. Clin Radiol.

[27] Merzench H (2012). Paediatric CT scan usage and referrals of children to computed tomography in Germany-a cross-sectional survey of medical practice and awareness of radiation related health risks among physicians. BMC Health Serv Res.

[28] Brady Z, Cain TM, Johnston PN (2012). Justifying referrals for paediatric CT. Med J Aust.

[29] Arslanoglu A (2007). Doctors’ and intern doctors’ knowledge about patients’ ionizing radiation exposure doses during common radiological examinations. Diag Interv Radiol.

[30] Almohiy H (2014). Paediatric computed tomography radiation dose: a review of the global dilemma. World J Radiol.

[31] Heyer CM, Hansmann J, Peters SA, Lemburg SP (2010). Paediatrician awarness of radiation dose and inherent risks in chest imaging studies-a questionnaire study. Europ J Radiol.

[32] Naumann DN (2014). Radiation exposure during paediatric emergency CT: time we took notice?. J Pediatr Surg.

[33] Goske MJ (2010). Image gently: providing practical educational tools and advocacy to accelerate radiation protection for children worldwide. Semin Ultrasound CT MRI.

